# A simple measure with complex determinants: investigation of the correlates of self-rated health in older men and women from three continents

**DOI:** 10.1186/1471-2458-12-649

**Published:** 2012-08-13

**Authors:** Davina J French, Colette Browning, Hal Kendig, Mary A Luszcz, Yasuhiko Saito, Kerry Sargent-Cox, Kaarin J Anstey

**Affiliations:** 1 Centre for Research on Ageing, Health and Wellbeing, Australian National University, Canberra, ACT, Australia; 2 School of Primary Health Care, Monash University, Melbourne, VIC, Australia; 3 Faculty of Health Sciences, University of Sydney, Sydney, NSW, Australia; 4 School of Psychology, Flinders University, Adelaide, South Australia, Australia; 5 Advanced Research Institute for the Sciences and Humanities, Nihon University, Tokyo, Japan

**Keywords:** Self-rated health, Older adults, Australia, Japan, South Korea, United States of America

## Abstract

**Background:**

Self-rated health is commonly employed in research studies that seek to assess the health status of older individuals. Perceptions of health are, however, influenced by individual and societal level factors that may differ within and between countries. This study investigates levels of self-rated health (SRH) and correlates of SRH among older adults in Australia, United States of America (USA), Japan and South Korea.

**Methods:**

Cross-sectional data were drawn from large surveys of older respondents (≥ 65 years) in Australia (n = 7,355), USA (n = 10,358), Japan (n = 3,541) and South Korea (n = 3,971), collected between 2000 and 2006. Harmonized variables were developed to represent socioeconomic, lifestyle and health indicators. We then assessed whether these variables, and their potentially different impact in different countries, could account for cross-national differences in levels of SRH.

**Results:**

SRH differed significantly between countries, with older Koreans reporting much poorer health than those in the other three nations. This was not the result of biases in response patterns (for example central versus extreme tendency). Health-related correlates of SRH were similar across countries; those with more medical conditions, functional limitations or poor mental health gave poorer ratings. After accounting for the differential impact of determinants in different national contexts, Australians reported better SRH than other nations.

**Conclusions:**

We conclude that when examining correlates of SRH, the similarities are greater than the differences between countries. There are however differences in levels of SRH which are not fully accounted for by the health correlates. Broad generalizations about styles of responding are not helpful for understanding these differences, which appear to be country, and possibly cohort specific. When using SRH to characterize the health status of older people, it is important to consider earlier life experiences of cohorts as well as national and individual factors in later life. Further research is required to understand the complex societal influences on perceptions of health.

## Background

Self-rated health (SRH) is used worldwide to assess health status with a single self-report item [1]. It is considered by the World Health Organization (WHO) to be an important indicator of population health and healthy life expectancy, due to the finding that self-rated health predicts major health outcomes including health care utilization, physical and functional health status, stroke, nursing home entry and even mortality [2]. Even when different questions and response options have been used, these relationships remain robust [3]. In contrast, studies seeking to understand the meaning of individual responses to questions such as ‘How is your health in general’ are in their infancy. Jylhä (2009) has offered a conceptual model to aid understanding of this issue, suggesting that the process by which individuals answer questions of this kind might involve them reviewing relevant facts about their own health (for example medical diagnoses, symptoms and functional limitations), making a comparison with some other reference group, and then deciding which of the available response options (typically excellent, very good, good, fair or poor) is the best match for their evaluation. Each element of the process takes place within a cultural context, suggesting that responses may not have the same meaning in different countries, cultures or cohorts. Individuals’ current and dispositional mood provide further context for evaluations.

### National and cultural differences in self-reports of health

A number of studies have compared SRH between countries or cultural groups. Such studies have investigated whether apparent differences in SRH can be attributed to individual differences in objective health and social indicators, or remain when these have been taken into account, termed the ‘residual regression’ approach [4]. Signs and symptoms of illness may also carry different meaning in different cultural contexts; when this is the case health indicators interact with country in the regression. Differences that remain after accounting for these interactions may then be attributed to differing interpretations of the question or rating scale in different countries, usually described as national or cultural differences in reporting behavior. Some studies have also investigated the comparability of these ratings using responses to vignettes to anchor SRH responses [4,5]. However, such a methodology may place too large a burden on respondents and is not feasible for population-based studies or when using existing datasets [6].

Using the regression approach, middle-aged and older Italians were shown to report poorer SRH than a French sample of the same age, but this difference was no longer significant when differences in socioeconomic factors, disease and disability were added to the equation [7]. By contrast, older Russians reported poorer SRH compared to Finns, a difference that remained when health and social indicators were accounted for [8]. Lee and Shinkai (2003) showed that after accounting for health and social indicators, the SRH advantage for Japanese elders over their Korean counterparts was reversed.

A number of other studies have investigated similar questions within European [9,10] and Asian [11] countries; all have established the importance of socioeconomic factors, chronic illnesses, functional limitations and mental health as determinants of SRH. Some studies have also identified a role for lifestyle factors, for example smoking [12], although these relationships differ between cultures [10]. Studies comparing respondents from widely differing cultural backgrounds, for example Anglo-Americans or Europeans versus Asians, have been limited to comparisons of cultural groups within Western nations [13], and these studies taken together offer no consensus about the factors that may account for different responding patterns in different countries.

### Explanations for differences in self-rated health

Some researchers have proposed that national differences in self-reports can be explained by differences in responding styles between Western and Asian groups. For example, it has been proposed that systematic cultural pressures towards self-enhancement in United States of America (USA) and self-criticism in Japan may underlie self-evaluations in these countries [14]. Culture may also influence willingness to use the full set of available response options [15]. A preference for the mid-point of the SRH scale has been noted in a sample of Japanese elderly, but these authors did not compare their data to a non-Asian sample [16].

While cultural differences between Eastern and Western countries may play a role, Jylhä’s model would also suggest that life history and personal circumstances are likely to influence responses. These may operate at the individual and the cohort level. For example, traumatic early experiences may result in increased psychological distress, or even depression, in later life which may provide a context for reporting SRH [17,18]. Such historical influences may be especially relevant for the older cohorts studied here, whose prior experiences include the Great Depression and World War 2. Ratings of health may also be influenced by current stress, for example changes in SRH over time within Japan have been related to a change in economic conditions brought about by the Asian financial crisis [19].

### Gender differences in self-rated health

Gender may provide a further context for individual SRH ratings, for example Lee and Shinkai (2003) and Lindeboom and van Doorslaer (2004) report gender differences in SRH responses in East Asian and Scandinavian samples respectively. Gender effects are not however found consistently within Europe [20], and were not apparent in a recent Australian study of older adults [21]. The influence of gender in relation to illness and illness-reporting is likely to be culturally and possibly historically determined, so it is particularly important for a study such as ours to consider whether, when objective health is controlled, SRH differs for men and women, and whether this gender difference varies cross-nationally.

### Aims of the study

Our study is the first to investigate cross-national differences in self-rated health between multiple Western and East Asian samples. We will first describe the levels of SRH in these samples and examine whether systematic differences in patterns of extreme versus mid-point responding are apparent. Comparison of the response patterns of samples from two Western and two East Asian cultures will allow us to address more directly the question of whether broad cultural differences in responding behavior can account for differences in SRH between countries. We will then use the residual regression approach to account for a range of individual health and social indicators; if cross-national differences in SRH remain, it is likely that country provides a context that influences SRH reports in addition to actual health. Interactions between country and individual indicators will assess whether this influence operates by changing the relationship between health indicators and SRH. We will also assess whether gender provides a context within which illness-related variables carry different weight by examining interactions between gender and health indicators. If cross-national differences in SRH remain in the presence of these main and interaction effects, we will be able to establish whether such differences are sample specific, or fit a pattern consistent with previous suggestions of a broad cultural difference between Western and East Asian responding styles.

## Methods

### Samples

Data were drawn from large surveys conducted between 2000 and 2006. The Japanese survey included only those aged 65 and over, therefore only responses from people aged 65 and older were selected from the other datasets although their original samples comprised those aged over 45 (Australia and Korea) and 50 (USA) years. Since SRH is subjective we also excluded responses from proxy informants. Each of the original studies received appropriate ethical approval while our study analyzed only de-identified data files. Our team included principal investigators responsible for the Australian (Anstey, Browning, Kendig, Luszcz) and Japanese (Saito) studies. Data from the United States and Korean surveys were available as public use datasets, which were obtained through registration with the relevant organizations (The University of Michigan Institute for Social Research and the Korea Labor Institute respectively).

#### Australia

The Australian data were drawn from the Dynamic Analyses to Optimize Ageing (DYNOPTA) dataset, which has pooled and harmonized data from nine Australian longitudinal studies of ageing [22]. Cross-sectional data from surveys collected between 2000 and 2006 were selected so as to maximize the available variables and sample size. Three contributing studies had all of the necessary variables hence the DYNOPTA sample for this study comprised 7,355 older Australians. The largest contributing study, the Australian Longitudinal Study of Women’s Health, used a national sample and a mail-out survey with an initial response rate of 40% [23]. The two smaller studies collected data in Adelaide, South Australia (Australian Longitudinal Study of Ageing) and in the Australian Capital Territory and surrounding region (Personality and Total Health through life study) using face-to-face interviews, with participation rates of 55% and 58% respectively [24,25]. Data were drawn from waves collected between 4 and 9 years after baseline; retention rates to the relevant wave for non-deceased participants ranged from 68 to 94% [22]. The proportion of women was very high (84%) since the largest study was of women’s health, see Table 1. 

**Table 1:** Demographic and health characteristics for each sample

**Characteristic**	**Australia**	**USA**	**Japan**	**Korea**
N	7,355	10,358	3,541	3,971
Sex (% male)	16.1	41.9	44.2	41.4
Mean age in years (SD)	77.2 (6.50)	74.5 (7.30)	74.4 (6.60)	73.0 (6.31)
Partner status (% partnered)	52.2	58.8	64.4	62.8
Educational level (%)				
Grade 9 or lower	27.3	16.6	60.6	82.1
Grades 10-12	37.8	46.3	29.1	11.7
Post-secondary	34.9	37.0	10.3	6.2
Smoking (% non-smoker)	95.0	90.2	83.0	84.8
Drinking Alcohol (% abstainer)	27.7	70.3	66.1	74.3
Medical conditions (%)				
None	19.3	7.7	32.5	36.3
One	32.8	24.9	45.1	39.3
More than one	47.9	67.4	22.4	24.4
ADL Difficulty (% with no difficulty)	86.4	86.1	96.9	91.3
Mental Health Status (% poor)	12.4	14.9	13.3	23.1
Self-rated health (%)^a^				
Poor	3.0	8.5	2.5	40.6
Fair	22.1	22.7	20.8	30.9
Good	40.4	32.4	41.2	23.5
Very good	27.3	27.6	19.1	4.0
Excellent	7.1	8.8	16.4	1.0
Mean self-rated health (SD)^b^	2.87 (0.94)	2.94 (1.09)	2.74 (1.04)	4.06 (1.11)

^a^ In the Japanese survey response options were totally healthy/relatively healthy/of average health/relatively not very healthy/not at all healthy.

^b^ Low scores indicate good health; excellent = 1 to poor = 5.

Note: Percentages may not sum to 100 due to rounding. All characteristics are significantly different between countries, *p* < .001 determined by Chi squared or F-test.

#### United States of America

The USA sample was drawn from wave 8 of the Health and Retirement Study (HRS) collected in 2006 [26]. HRS is a nationally representative survey of older Americans sponsored by the National Institute of Aging (grant number NIA U01AG009740) and has been conducted by the University of Michigan every two years since 1992 with periodic addition of younger cohorts. Initial response rates ranged from 86-89% across waves with wave-to-wave retention being better than 90% [27]. Data are collected by computer assisted interview; these are mainly by telephone but face-to-face interviews are preferred for the oldest respondents. Data for all relevant variables were available for 10,358 individuals.

#### Japan

Japanese data were from the second wave of the Nihon University Japanese Longitudinal Study of Aging (NUJLSOA), collected in 2001. Respondents were aged 65 years and older and were a representative national sample. The survey instrument was designed with reference to its comparability with HRS and administered by face-to-face interview. The survey achieved a 74.6% response rate at wave 1 with 81% of these respondents retained to wave 2; 65–66 year olds were also added to the wave 2 sample [28]. Complete data were available for 3,541 respondents.

#### Korea

Data for the Korean sample were drawn from the first wave of the Korean Longitudinal Study of Ageing (KLoSA), conducted by the Korea Labor Institute (KLI) in 2006. The sample represented the population of Korean elders except for residents of Jeju Island. The household response rate was 70.7% at wave 1 [29]. Complete data were available for 3,971 respondents aged 65 years and over. The KLoSA survey was designed to be comparable with similar panel studies, such as HRS. The KLoSA survey instrument was administered with computer assistance during a face-to-face interview.

### Materials and data harmonization

#### Sample characteristics

Year of birth and sex were available in all datasets. Questions on current marital status were harmonized to produce a binary variable denoting whether the respondent was currently living with a partner or not. The only variable indicating socioeconomic advantage that was available in all four surveys was educational attainment. This variable used different but partially overlapping response options in the four countries, which reflected their somewhat different educational systems. We retained the maximum information by using a harmonized three category variable of ‘schooling to Grade 9 or less’ (which included a substantial proportion with no formal education in the Asian samples), ‘completed school to Grades 10, 11 or 12’ and ‘post-secondary education’, which included both 2-year diplomas and tertiary degrees.

#### Health behavior

Data from all four surveys allowed us to categorize respondents as ‘never smoker’, ‘former smoker’ or ‘current smoker’, however the variable used in analysis collapsed never and former smokers since the low proportion of female former smokers in Korea prevented analysis of this category separately. All respondents were asked whether they ever drank alcohol. Those who responded ‘no’ were classified as non-drinkers. For drinkers, frequency and quantity of drinking were available, however preliminary analyses demonstrated that only the difference between drinking or not was associated with SRH so the binary variable ‘drinker’ versus ‘abstainer’ was used in analyses.

#### Health indicators

Self-reported medical conditions were recorded in all four surveys although the diseases varied. Questions about diabetes, cardiovascular conditions (including high blood pressure), chronic respiratory conditions, arthritis and cancers other than skin cancer were common to all countries. Together with mental illness, which we have included here as a separate variable, these conditions are responsible for most of the disease burden among older Australians, with the only other major contributors being dementia, which is likely to have precluded participation in the studies, and adult hearing loss [30]. We collapsed responses to give a variable describing whether respondents had reported having none, only one, or more than one of the five disease groups listed above.

Self-reported disability was reported more extensively in some surveys than others. Our analysis used the only functional limitation questions common to all countries: whether or not respondents reported any difficulty or limitation in bathing, showering or dressing. All surveys included a measure of psychological health. The USA, Japanese and Korean surveys each used a version of the Centre for Epidemiological Studies - Depression Scale (CES-D) [31] however they each used different items and response scales. The DYNOPTA studies that we analyzed used either the standard CES-D 20 or Mental Component Scores (MCS) from the SF-36 [32].

We harmonized these variables by coding participants as having probable depressive symptomatology if they met cut-offs previously established as equivalent to the common threshold of ≥16 used for the CES-D 20. Thresholds were drawn from studies using samples of similar age and cultural background, and where possible from previous calibrations of the datasets we have employed. HRS used an 8-item CES-D with binary responses; cross validation has recommended a cut-off ≥4 for this scale [33]. The Japanese survey used 11 items and a three point response scale [34], with a cut off ≥7 recommended for this sample [35].

The Korean survey used the CES-D 10 developed for use with elderly respondents [36], and a standard 4 point response scale. In the absence of sample-specific recommendations, we consulted a broader research literature to reach a best estimate for our sample. Two studies recommended a CES-D 20 cut-off higher than ≥16 (≥21 and 22) when used with Korean elderly [37,38]. A cut off ≥22 for the CES-D 20 has also been suggested for Hong Kong elderly, in a study which also recommended ≥12 for the CES-D 10 [39]. We therefore adopted ≥12 as the most appropriate cut-off for our sample. For the Australian data we used ≥16 for the study using the CES-D 20 and ≤42 as the equivalent value for establishing probable depressive illness on the MCS [40]. Since this harmonized variable does not provide a diagnosis we refer to the groups created as those in ‘poor mental health’ and ‘good mental health’.

#### Self-rated health

All four surveys measured SRH; HRS, DYNOPTA and KLoSA asked *‘Would you say your health is…’* followed by five response options labeled ‘excellent’, ‘very good’, ‘good’, ‘fair’ and ‘poor’. NUJLSOA asked *‘In general, how would you describe your state of health?’* but the five response options were more accurately translated as ‘totally healthy’, ‘relatively healthy, ‘of average health’, ‘relatively not very healthy’ and ‘not at all healthy’.

### Statistical analysis

Correlates of SRH were examined using the generalized linear modeling procedure in SPSS19.0. An ordinal probit model was selected since SRH was distributed normally in the pooled data (skewness = −0.01, SE 0.02). Odds ratios show the change in odds of reporting poorer health. We first tested a model including all socio-demographic and health variables (model 1). All were categorical except for age. Reference groups were; male, partnered, lowest educational attainment, non-smoker, abstainer, no medical conditions or disability and good mental health. In the second model a variable representing country was added, with Korea as the reference category, and the third model tested interactions between country and each of the variables in model 1. The nature of significant interaction effects was further explored by repeating the analyses separately for each country to compare the size and direction of the odds ratios obtained. A further model tested interactions between gender and each of the model 1 variables. Where multiple pair wise tests were undertaken to examine between-country differences, alpha levels were reduced using a Bonferroni correction. This always involved a set of six comparisons, reducing alpha to .0083. While the Bonferroni approach is often considered overly conservative, it is unlikely to introduce unwanted type II error in our very large samples.

## Results

All of the characteristics reported in Table 1 exhibited significant differences between countries. The Australian sample was older, more likely to be female, and less likely to be partnered than the other samples and education levels were higher in Western than Asian countries. Australian and USA respondents were more likely to report medical conditions and ADL difficulties than their Asian counterparts. Poor mental health was considerably more prevalent in the Korean sample despite our precaution of using a higher threshold.

### Cross-national differences in self-rated health

Self-rated health differed markedly between countries; follow-up pair wise comparisons showed that all between-country differences were statistically significant however the largest difference was clearly between Korea and the other three nations. In Australia, USA and Japan the distribution of SRH was approximately normal and ‘good’ or ‘average’ health was the most common response. The proportion of responses in the ‘good’ or ‘average’ category was very similar in Japan and Australia, suggesting that mid-point preference is not a specifically Japanese phenomenon. In Korea the distribution of responses was highly skewed with ‘poor’ health the most common response. The largest proportion of responses in the excellent category, and the lowest mean score, was in the Japanese sample. A formal test of the hypothesis that East Asians avoid extreme response options was not undertaken since it is clear from these data that each of the extreme options was used most frequently in an Asian country.

### Gender differences in self-rated health

Table 2 shows SRH separately for men and women in each country. Ratings were lower (healthier) for men than women in all countries. It is apparent that the lower SRH values for men in Australia are due to their ratings tending towards the very good/excellent end of the scale, while women tended towards the centre; few Australians of either gender reported poor health. In contrast, the high proportion of poor ratings in Korea is especially noticeable for older Korean women, for whom ‘poor’ was the most frequently used response.

**Table 2:** Self-rated health (SRH) for men and women in Australia, USA, Japan and Korea

	**Australia**^**a**^		**USA**^**a**^		**Japan**^**ab**^		**Korea**^**a**^	
	**Male**	**Female**	**Male**	**Female**	**Male**	**Female**	**Male**	**Female**
N	1,182	6,173	4,343	6,015	1,564	1,977	1,644	2,327
SRH (%)								
Poor	2.2	3.2	7.5	9.2	2.7	2.2	30.9	47.4
Fair	10.5	24.3	22.3	23.0	17.3	23.7	31.0	30.9
Good	30.3	42.3	32.6	32.2	41.6	40.9	31.1	18.0
Very good	40.9	24.7	27.8	27.4	19.2	19.0	5.9	2.7
Excellent	16.1	5.4	9.8	8.1	19.1	14.2	1.1	0.9
Mean SRH^c^	2.42	2.95	2.90	2.98	2.65	2.81	3.84	4.21
(SD)	(0.95)	(0.91)	(1.09)	(1.09)	(1.06)	(1.02)	(0.97)	(0.89)

^a^ Without covariates, men reported significantly better health than women in all countries, *p* < .001.

^b^ In the Japanese survey response options were totally healthy/relatively healthy/of average health/relatively not very healthy/not at all healthy.

^c^ Low scores indicate good health; excellent = 1 to poor = 5.

Note: Percentages may not sum to 100 due to rounding.

### Correlates of self-rated health

The odds ratios and significance tests from ordinal regression analyses examining correlates of SRH are shown in Table 3. Increased likelihood of poorer SRH was associated with being male, less educated, being a smoker or abstainer and with reporting more medical conditions, functional disability or poor mental health. If differences in these variables account for cross-national differences in SRH then the country variable should be non-significant when added in model 2. The odds ratios however show that country is strongly associated with self-ratings of health, with the likelihood of poor ratings very much reduced in other countries compared to Korea. After adjusting for multiple comparisons, all countries differed from each other except USA and Japan.

**Table 3:** **Odds ratios**^**a**^**for correlates of poorer self-rated health**

**Predictor (reference group)**	**Model 1**		**Model 2**	
	**OR (95 % CI)**	**Model effect*χ***^**2**^**(DF)**	**OR (95 % CI)**	**Model effect*χ***^**2**^**(DF)**
Gender (male)	0.97 (0.94-0.99)	5.41_(1)_*	1.01 (0.98-1.04)	0.37_(1)_
Age (years)	1.00 (1.00-1.00)	0.10_(1)_	1.01 (1.01-1.01)	54.82_(1)_***
Partner status (partnered)	1.03 (1.00-1.05)	3.16_(1)_	1.05 (1.02-1.08)	12.71_(1)_***
Education (Grade 9 or lower)		2058.55_(2)_***		428.36_(2)_***
Grades 10-12	0.58 (0.56-0.60)		0.81 (0.78-0.84)	
Post-secondary	0.46 (0.44-0.48)		0.66 (0.63-0.69)	
Smoking (non-smoker)	1.18 (1.12-1.23)	50.63_(1)_***	1.17 (1.12-1.23)	47.92_(1)_***
Alcohol (abstainer)	0.77 (0.75-0.80)	331.24_(1)_***	0.80 (0.78-0.83)	194.80_(1)_***
Medical conditions (none)		1080.16_(2)_***		2075.39_(2)_***
One	1.36 (1.30-1.41)		1.59 (1.53-1.66)	
More than one	1.83 (1.76-1.90)		2.51 (2.41-2.61)	
ADL difficulty (none)	1.99 (1.90-2.08)	935.02_(1)_***	2.19 (2.09-2.29)	1154.01_(1)_***
Mental health (good)	2.14 (2.06-2.23)	1473.45_(1)_***	2.13 (2.04-2.21)	1381.77_(1)_***
Country (Korea)				4266.66_(3)_***
Australia			0.24 (0.23-0.26)	
USA			0.22 (0.21-0.23)	
Japan			0.22 (0.21-0.23)	

*p < .05; ***p < .001.

^a^ Odds ratios are shown with 95% confidence intervals.

In the third analysis all of the interactions between country and the model 1 variables were added to the model and all were significant (*p* < .001). This model is not reported in Table 3 since the odds ratios for the ‘main’ effects cannot be interpreted in the same way as those for the preceding models. After accounting for potential correlates and their interactions, the likelihood of poorer SRH still differed significantly between countries, although the size of the model effect (*χ*^2^) was considerably reduced (from 4266.7 to 54.28, both *p < .001)*. In the presence of interaction terms, odds ratios cannot be used to indicate where the between-country differences lay, so a series of six analyses was carried out, testing all possible pair wise combinations of countries with all other model 3 variables and interactions. In these analyses, the significance of the model effect for country provides an indication of which countries differ in model 3. They showed that in Australia self-rated health was evaluated more positively than in all other countries, but the remaining three countries did not differ from each other using the Bonferroni corrected alpha level *p* < .008.

### Cross-national and gender differences in the correlates of self-rated health

To further understand the interactions we conducted a set of regressions for each country separately; these are reported in Table 4. Gender was independently associated with self-ratings of health only in USA and Korea and these effects were in opposite directions; the likelihood of poorer SRH was decreased for females in USA (OR 0.92, 95% CI 0.88-0.96), but increased for females in Korea (OR 1.18, 95% CI 1.07-1.30). Older age and lower educational attainment increased the likelihood of poorer ratings in all countries except Japan. Abstaining from alcohol was associated with increased likelihood of poorer SRH in all countries except Korea, while the effect of being a smoker was significant only in the Western countries.

**Table 4:** **Odds ratios**^**a**^**for correlates of poorer self-rated health in Australia, USA, Japan and Korea**

**Predictor (reference group)**	**Australia**	**USA**	**Japan**	**Korea**
Gender (male)	1.02 (0.94-1.11)	0.92 (0.88-0.96)***	0.99 (0.91-1.08)	1.18 (1.07-1.30)**
Age (years)	1.03 (1.02-1.03)***	1.00 (1.00-1.01)*	1.01 (1.00-1.01)	1.01 (1.01-1.02)***
Partner status (partnered)	1.08 (1.03-1.14)**	1.09 (1.04-1.14)***	1.06 (0.97-1.16)	0.85 (0.78-0.93)***
Education (Grade 9 or lower)				
Grades 10-12	0.91 (0.86-0.97)**	0.72 (0.67-0.76)***	0.92 (0.88-1.00)	0.68 (0.61-0.76)***
Post-secondary	0.78 (0.73-0.84)***	0.57 (0.54-0.61)***	0.92 (0.82-1.04)	0.57 (0.50-0.67)***
Smoking (non-smoker)	1.32 (1.17-1.48)***	1.33 (1.24-1.42)***	1.02 (0.92-1.13)	0.97 (0.87-1.08)
Alcohol (abstainer)	0.85 (0.80-0.90)***	0.77 (0.74-0.81)***	0.79 (0.73-0.88)***	0.93 (0.85-1.02)
Medical conditions (none)				
One	1.61 (1.50-1.73)***	1.58 (1.45-1.72)***	1.68 (1.55-1.83)***	1.56 (1.44-1.70)***
More than one	2.58 (2.40-2.78)***	2.66 (2.45-2.88)***	2.33 (2.10-2.58)***	2.38 (2.15-2.63)***
ADL difficulty (none)	2.26 (2.09-2.44)***	2.11 (1.98-2.25)***	2.46 (1.99-3.05)***	2.84 (2.38-3.38)***
Mental health (good)	2.32 (2.14-2.51)***	1.94 (1.83-2.07)***	1.91 (1.71-2.12)***	2.59 (2.34-2.86)***

*p < .05; **p < .01; ***p < .001.

^a^ Odds ratios are shown with 95% confidence intervals.

While the effect of medical conditions was shown to interact with country, inspection of the odds ratios revealed this effect to be in the same direction and of similar magnitude in all countries. Functional disability, although somewhat less common in Japan and Korea, exerted a slightly larger effect on SRH in these countries. Reporting poor mental health was associated with a marked increase in the likelihood of poor SRH in all countries, but this effect was somewhat larger in Australia and Korea, where the 95% confidence intervals did not overlap with those for the other two countries.

A test of the interactions between gender and the model 1 variables revealed that none of the gender interactions were significant; this analysis is not reported further.

## Discussion

Perhaps the most striking finding from this study is the similarities in the health-related correlates of SRH reported in Table 4. Individuals with more medical conditions, functional limitations or poor mental health gave poorer ratings in all countries and these factors accounted to some degree for the cross-national differences in raw SRH scores. However, some country level differences remained after accounting for these individual health correlations, and the respondents’ country provided a context within which some correlates carried different levels of influence.

Seeking explanations for these cross-national differences requires consideration of complex historical, economic and cultural factors that potentially influence individuals’ health and health perceptions. These wider influences potentially extend beyond those located at the individual level (e.g. educational background) and extend to national (e.g. health service availability), cultural (e.g. gender expectations) and cohort (e.g. experiences of war and economic depression) levels. Further, these various factors are likely to interact. For example, older Japanese and Korean men may share a cultural acceptance of cigarette smoking, but their childhood and young adult experiences (in oppressor and oppressed nations respectively) differ both from each other and from ascendent cohorts now at younger ages. Similarly, whether or not one is married is an individual level indicator, but its impact may depend upon cultural and economic circumstances that vary at the societal level.

### Cross-national differences in self-rated health

When considering the raw ratings, older Koreans rated their health as very much poorer than those in the three other countries. They were also more likely to report poor mental health and, further, the associations of mental health and functional disability with SRH were somewhat larger in Korea than other countries. The inclusion of social and health variables, along with their interactions, left Korean ratings differing only from those provided by the more highly rated Australian sample, suggesting that for Korean elders poor SRH largely reflects poorer objective health and its greater impact. Several factors might combine to explain these findings, although without access to relevant data to test these ideas, interpretation of the findings remains speculative. Firstly, this cohort of older Koreans may be uniquely vulnerable to poor health, and especially poor mental health, possibly as a result of their early life experiences. During the first half of the twentieth century, Korea suffered a particularly troubled history, with the greatest pressures on the population falling during the period 1935–1945 [41]. Most of our respondents were children or teenagers for all or part of this decade, with the youngest having been born in 1941; such difficult circumstances early in life could have had a lasting effect on their mental health and the way they perceive their lives. Such an effect could be specific to Koreans of this cohort. Future research should attempt to separate age from cohort effects in Korea, preferably through cross-sequential analyses or by examining a later cohort (e.g. those 45 to 64 years) who would have not experienced the same levels of deprivation early in life. This could clarify the relative importance of the historical influence discussed above as well as the economic factors associated with ageing and retirement considered below.

Secondly, although the four countries studied here are currently considered to have very high levels of human development [42], the present economic circumstances of older people in these countries may not be equivalent. It is possible that the very rapid and later social improvements in Korea, which contrast with earlier improvements in population level economic well-being in the other countries, may not have benefited older people as much as younger people [43]. A related factor may be emergent tensions in filial expectations and supports. Traditional Korean family exchange has been ordered by the principles of Confucian philosophy, within which *Hyo*, the duty of children to care for their parents, is a core value [44]. The proportion of elderly Koreans living with their children was, however, considerably reduced by the end of the twentieth century [45]. At the time of our survey more Koreans (approximately 30%) remained in the labor force after 65 years of age than in Western countries, suggesting that they may not be receiving either the filial or public support that they might have expected [46].

Labor force participation is also high among men above the retirement age in Japan, notwithstanding the mature social security system that has provided better retirement income: previous studies have suggested that continuing to work serves social and psychological benefits for Japanese men [47]. Older Koreans, however, have had less access to retirement income and their jobs are more likely to be casual, low-skilled and poorly paid compared to younger workers [46]. Such conditions may further contribute to their poor mental health as they may continue to work out of economic necessity more than choice. Self reports of poor health by Korean elders appear to be largely accounted for by objective differences in their reported physical and mental health, and the ways in which those health conditions impact upon them, suggesting that they are a cohort in need of further study and potentially additional support. This picture of poor health in Korea is especially pronounced for women, suggesting that gender inequalities should also be a focus of further study.

After accounting for differences in social and health status, and the ways in which these factors operate differently in different countries, the remaining cross-national differences in level of SRH indicate that, *ceteris paribus,* older Australians provided more optimistic assessments of their health than older adults in the other countries. Systematic differences in preference for extreme versus central responding in Western versus Asian countries do not appear to account for this finding. The data in Table 1 suggest that preference for the extreme negative response option may be characteristic specifically of the Korean sample, although past research has hypothesized a *central* tendency underlying some Asian response patterns. Table 2 further shows that any extreme responding preference was in fact confined to women; Korean men used the good, fair and poor responses in approximately equal proportions. An extreme negative response set may partially account for our findings, but it is important to note that it is confined to Korean women, and is not amenable to any broader gender or cultural explanation. It seems likely that explanations may lie in contextual influences that are specific to countries and/or cohorts rather than in crude distinctions between Western and East Asian ways of thinking or responding. It is also notable that Australian ratings differ significantly from those in the USA, notwithstanding many social and cultural similarities. Future studies should include indicators of access to health care, for example provision of public health care and health insurance subsidies: the better access in Australia than the USA in both these respects may be important for individual perceptions of health.

The changing position of Japan in the ranking, after taking personal factors into account, is partly consistent with Lee and Shinkai’s (2003) findings – in our raw data mean SRH was also healthiest in Japan. After accounting for all other variables, Japan’s position was not reversed with respect to Korea, as it was in Lee and Shinkai’s study, but it became significantly worse than Australia and was no longer better than Korea. This suggests that the original ratings of better health in Japan were reported because the Japanese elders were indeed somewhat healthier, with the lowest reports of chronic illness and disability, so their ‘advantage’ in SRH was a real one and apparent differences were reduced once health indicators were was taken into account.

### Cross-national differences in the correlates of self-rated health

Cross-national differences notwithstanding, we wish to re-iterate that the health-related correlates of SRH were found to be similar across countries; those with more medical conditions, functional limitations or poor mental health gave poorer ratings. Our findings, in conjunction with the majority of past studies, support a conclusion that health-related correlates of SRH are found universally. Although cross-national differences in SRH were initially large, they were much reduced when these health-related factors, along with social factors and the differing contextual effects of these, were taken into accounted.

Potentially important differences in impact were observed for some social and behavioral variables, for example poorer SRH was associated with being a current smoker only in Australia and USA. In these countries cigarette smoking has declined significantly and they are now well into Stage IV of the ‘smoking epidemic’, where smokers are in a minority and smoking is regarded as socially abnormal and a major health risk [48]. In Japan and South Korea however, smoking rates are still much higher, especially among males and adults younger than those surveyed here. Health-related behaviors such as smoking may influence perceptions of health (independently of their indirect effect through their actual health impact) via the messages that are current in the individual’s social climate. This could have the paradoxical effect of producing poorer health ratings among older smokers in nations where health promotion is more effective and risk behaviors are less prevalent.

A recent review has suggested that there is little health inequality associated with socioeconomic disadvantage in Japan, and that the longevity of Japanese elders is associated with a range of factors specific to Japanese culture [49]. We also found that none of the socioeconomic indicators were significant correlates of SRH in Japan. Data from the same NUJLSOA survey have also shown that educational attainment was unrelated to a more objective health indicator, functional disability [50]. These authors proposed that education may not be necessary to maintain good health in Japan, since access to health care is universal. These findings reinforce our contention that the impact of individual level indicators is moderated by national factors. Such complex effects can be revealed by cross-national research such as ours, complemented by examining possible changes after major economic or policy change [19,51]. It is perhaps worth noting that the beneficial effect of education, although significant, was also smaller in Australia than the remaining two countries; this may also be explained by a strong system of universal health care.

Although none of the other correlates of SRH showed an interaction with gender, we found differences in the effect of gender between nations. After taking differences in socioeconomic factors and health indicators into account, women were more optimistic in the USA (consistent with Grol-Prokopczyk and colleagues’ (2011) finding using a vignette approach) and more pessimistic in Korea than their male counterparts, while in Australia and Japan no gender difference was observed. This contrasts with the pattern in the raw data (Table 2) which showed poorer SRH among women in all countries. In Australia and Japan this gender difference in ratings disappeared after accounting for differences in indicators of health status (diagnoses, physical limitation and depressive symptoms), suggesting that the observed gender differences in SRH reflect gender differences in health in these countries. In the USA the difference was actually reversed in multivariate analysis, with women’s ratings being more positive than men’s after accounting for illness burden. Only in Korea did women report their health more negatively than men after accounting for their (also higher) health burden. Our data highlight the fact that gender is a social construction that resides within a wider culture, and which is a stronger correlate in some countries than others. Future researchers should remain aware that the effects of both gender and education may be specific to the sample under investigation.

### Limitations

Our study is based on large and representative samples, and our selection of countries allows us to draw valuable inferences about the meaning of SRH responses. However, the process of making cross-national comparisons based on secondary data also has limitations. Firstly, there is inherent difficulty in establishing whether the Korean and Japanese surveys convey exactly the same meaning to respondents as the English questionnaires. While translations were undertaken with great care, we should remain aware that some constructs may have different meanings in different languages and cultures. In particular the response scale for the SRH question in the Japanese survey was worded somewhat differently which may have affected the distribution of responses in this country.

Secondly, several of the surveys employed complex sampling strategies, so the use of sampling weights may have been preferred. Weights were not, however, available for the Australian data so this approach could not be taken. Simulations have demonstrated that the use of population weights is, in any case, of dubious value for regression analyses such as those reported here, although our estimates of the levels of self-reported health may be affected [52].

Thirdly, we are mindful of particular limitations associated with our mental health variable – mental health is clearly an important determinant of SRH, but our indicators may be too variable to draw firm conclusions about how this effect varies between countries. We also note that the CES-D is primarily a depression measure. Some studies of older Holocaust survivors (e.g. Sharon et al., 2009) have found elevated anxiety but not depression; anxiety among survivors of other traumatic war-time experiences may also influence SRH and is not accounted for in our data.

Fourthly, we were limited by the lack of a more contemporaneous socioeconomic indicator; especially for older persons educational attainment may have been superseded by employment-related advantages or disadvantages, and may be less relevant for older people in societies undergoing rapid socioeconomic development. As discussed above, current financial stress may be an important factor in driving both mental health and SRH; a variable assessing income would be valuable to explore this idea further. More broadly, better measures of social class position are needed for examining social determinants of health in older people [53].

Fifthly, we are aware that survivor effects, both from birth and within the longitudinal waves of a study, may differ between the cohorts that we have used. The potential importance of survival effects can be illustrated by the fact that, even in the relatively advantaged cohort of Australians, only 75% of their birth cohort would have survived to age 65 years at the time of the survey [54]. Our decision to constrain the dates of the surveys to a single decade, in order to match the respondent’s historical cohort, has also resulted in the use of data collected in later waves in some surveys than others. Only the Korean data used responses to a wave one survey so it remains a possibility that respondents in other countries have been subject to a greater degree of selection since recruitment than the Korean sample.

Finally, because we were interested in *self-*rated health, we only used data from older adults who were sufficiently well (physically, emotionally and cognitively) to participate in the surveys themselves; our findings may not generalize to the very sick and institutionalized elderly. In light of our speculation that historical effects may be important for these samples, we also recommend caution in generalizing our findings to younger cohorts.

## Conclusions

This study presents a number of methodological and scientific challenges for global research in health and ageing. In order to progress cross-national research on these issues, there is a need to develop multi-country teams of researchers prior to collecting data, to ensure the optimal comparability of measures and the inclusion of social and health indicators that are validated and robust in various countries. We also advocate the inclusion of life history measures to aid in the development of more informed explanations of the social resources, vulnerabilities, and exposures that influence health outcomes in later life within and between country differences; to date life history studies have been limited to Western countries [55]. For better understanding of the meanings of self rated health, there is promise in applying the vignette approach including vignette calibration studies that may need to be repeated many times in different countries and cohorts. It is also important to examine broader dimensions of cultural differences, for example, the influence of Protestant religions in the USA in contrast with Buddhism in Eastern countries [56]. The inclusion of biomarkers to validate self-reported health indicators should also be a priority.

We conclude that, at least when considering self-ratings of health, broad East versus West generalizations are over-simplistic – while the overall predictors of SRH were similar across the four countries we studied, there were also differences between countries that are quite specific. These were not due to a simple reporting effect, but indicated contextual differences in the importance of particular variables (for example gender, smoking) for understanding SRH responses in different countries. Researchers should therefore be cautious of making broad statements about how cultural groups ‘respond’ collectively, and instead consider differing historical and social contexts within and between countries. In light of the power of SRH to predict mortality over and above health indicators, we consider these contexts to be far from ‘artefactual’ but to provide important information about the meaning and impact of health for those who report it.

## Competing interests

The authors declare that they have no competing interests.

## Authors’ contributions

CB, HK, MAL and KJA participated in conception and data harmonization for the DYNOPTA study. YS participated in conception, design and data collection for the NUJLSOA study. DJF conducted the cross-national harmonization and statistical analysis and drafted the manuscript. All authors participated in the design of the current study, interpretation of the results and made critical contributions to the manuscript. All authors read and approved the final manuscript.

## Pre-publication history

The pre-publication history for this paper can be accessed here:

http://www.biomedcentral.com/1471-2458/12/649/prepub
